# PFDB: A standardized protein folding database with temperature correction

**DOI:** 10.1038/s41598-018-36992-y

**Published:** 2019-02-07

**Authors:** Balachandran Manavalan, Kunihiro Kuwajima, Jooyoung Lee

**Affiliations:** 1 School of Computational Sciences, Korea Institute for Advanced Study (KIAS), Seoul, Korea; 2 CPIS, the Graduate University for Advanced Studies (Sokendai), Hayama, Japan; 3 Department of Physics, School of Science, the University of Tokyo, Tokyo, Japan

## Abstract

We constructed a standardized protein folding kinetics database (PFDB) in which the logarithmic rate constants of all listed proteins are calculated at the standard temperature (25 °C). A temperature correction based on the Eyring–Kramers equation was introduced for proteins whose folding kinetics were originally measured at temperatures other than 25 °C. We verified the temperature correction by comparing the logarithmic rate constants predicted and experimentally observed at 25 °C for 14 different proteins, and the results demonstrated improvement of the quality of the database. PFDB consists of 141 (89 two-state and 52 non-two-state) single-domain globular proteins, which has the largest number among the currently available databases of protein folding kinetics. PFDB is thus intended to be used as a standard for developing and testing future predictive and theoretical studies of protein folding. PFDB can be accessed from the following link: http://lee.kias.re.kr/~bala/PFDB.

## Introduction

Protein folding is one of the most difficult problems in biophysics and molecular biology. Due to the accumulation of over half a century’s experimental data on reversible folding-unfolding mechanisms^1,2^, at least 16 protein folding kinetics datasets have been reported^3–19^. However, there are many problems in these datasets, including variations in temperatures (from 5 °C to 75 °C) used in kinetic folding experiments, redundant data entries, and inadequate reported data. A more complete dataset of protein folding kinetics with corrections for the above problems is thus required, and once we have such a dataset, it will be very useful for developing and testing future predictive and theoretical studies of protein folding.

Here, we thus carefully examined the existing protein folding datasets, and introduced the necessary corrections. Among the available datasets, ACPro^19^ and the dataset by Garbuzynskiy *et al*.^17^ (hereinafter referred to as the Garbuzynskiy dataset) were the most recent ones, which contained the most updated and largest entries. Therefore, we utilized these two datasets in the current study to construct a new database called PFDB. Furthermore, we added new protein data into the PFDB from our own collection based on extensive literature search, which resulted in the entry size of 141 globular proteins in our dataset; whose size is the biggest among the currently available protein folding datasets.

In this study, we also developed a new temperature correction method for the proteins whose kinetic folding and unfolding experiments had been carried out at a temperature different from the standard temperature (25 °C). Our temperature correction method is based on the Eyring–Kramers equation^20^, and the logarithmic rate constants of folding and unfolding, ln(*k*_f_) and ln(*k*_u_), respectively, at 25 °C is provided for all proteins in PFDB. Interestingly, the present study is the first to introduce the temperature corrections into the protein folding dataset, and we show that the introduction of the temperature correction has improved the quality of the database. PFDB is thus currently the most updated database of protein folding kinetics, and hence it can be used as a standard for developing future predictive and theoretical studies of protein folding.

## Results and Discussions

### Database construction and descriptions

We first combined the two most recent datasets of protein folding, the ACPro and Garbuzynskiy datasets, to construct the combined dataset (hereafter called “the AG dataset”) in which redundant or inappropriate entries were filtered out. We excluded the proteins containing disulfide linkages or covalently bound prosthetic groups, because the presence of these linkages or groups can significantly affect the folding kinetics. Small polypeptides with less than 34 residues were also excluded. We carefully examined each data in the AG dataset. For instance, if there is no updated protein folding kinetics data available for a protein, we included those proteins as such in PFDB, otherwise replaced with the updated data. Furthermore, we added the data of 33 new proteins into the PFDB from our own collection based on extensive literature search, resulting in the entry size of 141 globular proteins (89 two-state (2S) and 52 non-two-state (N2S) proteins) in our dataset (see Methods for details of the database construction).

Our dataset lists the following items: (i) the protein short name with a reference to the original experimental paper(s) on the folding kinetics, (ii) the PDB code, (iii) the structural class (α, β, α/β, and α + β), (iv) folds in the SCOP classification^21^ (http://scop.mrc-lmb.cam.ac.uk/scop/), (v) the number of residues in the PDB structure (*L*_PDB_), (vi) the actual number of residues of the protein used in the folding experiment (*L*), (vii) the experimental conditions (pH and temperature), (viii) the folding type (2S or N2S), (ix) the ln(*k*_f_) value reported, (x) the ln(*k*_f_) value after the temperature correction for the proteins whose folding experiments were carried out at a temperature other than 25 °C, (xi) the logarithmic rate constant of formation of a folding intermediate, ln(*k*_I_), when the value is available in the literature (only for N2S proteins), (xii) the ln(*k*_u_) value reported, (xiii) the ln(*k*_u_) value after the temperature correction, and (xiv) the Tanford *β* (*β*_T_) value, which is defined as *β*_T_ = 1 − (*m*_u_^‡^/*m*_NU_), where *m*_u_^‡^ (kJ/mol/M) and *m*_NU_ (kJ/mol/M) are the denaturant concentration dependence of the activation free energy of unfolding and the denaturant concentration dependence of the unfolding free energy from the native (N) to the fully unfolded (U) state, respectively^22^. The ln(*k*_f_), ln(*k*_I_) and ln(*k*_u_) values listed in PFDB are those in the absence of denaturant, usually obtained by linear extrapolation of the logarithmic rate constant along denaturant concentration.

**Figure 1:**
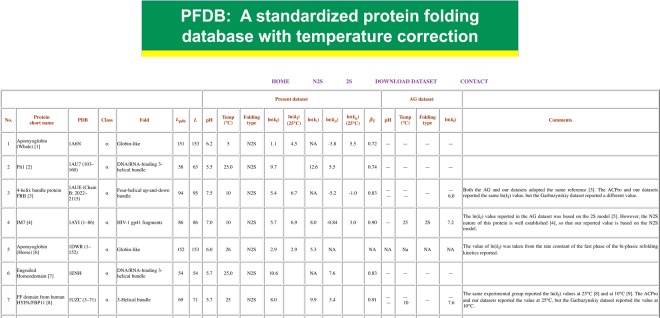
A snapshot of our dataset in the PFDB homepage. For each protein, our dataset lists (i) protein short name, (ii) PDB code, (iii) structural class (α, β, α/β, and α + β), (iv) folds in the SCOP classification, (v) the number of residues in the PDB structure (*L*_PDB_), (vi) the actual number of residues of the protein used in the folding experiment (*L*), (vii) experimental conditions (pH and temperature), (viii) folding type (2S or N2S), (ix) ln(*k*_f_) reported, (x) ln(*k*_f_) after temperature correction, (xi) ln(*k*_I_) (only for N2S proteins), (xii) ln(*k*_u_) reported, (xiii) ln(*k*_u_) after temperature correction, and (xiv) Tanford *β* (*β*_T_). The AG dataset is also included in our database for comparison. A comment section is provided in the final column.

In PFDB, the folding type is thus clearly specified. The proteins that exhibited a stable folding intermediate during the kinetic folding process were classified as N2S proteins, while the proteins, exhibiting the single-exponential kinetics of folding without stable intermediates, were classified as 2S proteins even if the existence of an unstable high-energy intermediate was expected from the unfolding-limb or the folding-limb curvature of the chevron plot^23^. To discriminate the 2S proteins with a high-energy intermediate from the other 2S proteins, the former proteins were denoted by 2S*. Each entry of the AG dataset is also included in PFDB for comparison. A comment section is provided in the final column of the dataset and interprets discrepancies between the present and the AG datasets if any/necessary. Figure 1 depicts a snapshot of our dataset shown in the PFDB homepage.

The protein composition in PFDB in terms of the folding type and the structural class is given in Table 1. It shows that both the 2S and N2S proteins cover all four structural classes of globular proteins. However, the 2S proteins contain only one α/β protein.

**Table 1:** The composition of the PFDB in terms of structural and folding class is shown.

Folding type	Structural class				
	α	β	α + β	α/β	Total
2S	24	39	25	1	89
N2S	10	13	16	13	52
Total	34	52	41	14	141

### Temperature correction

**Figure 2:**
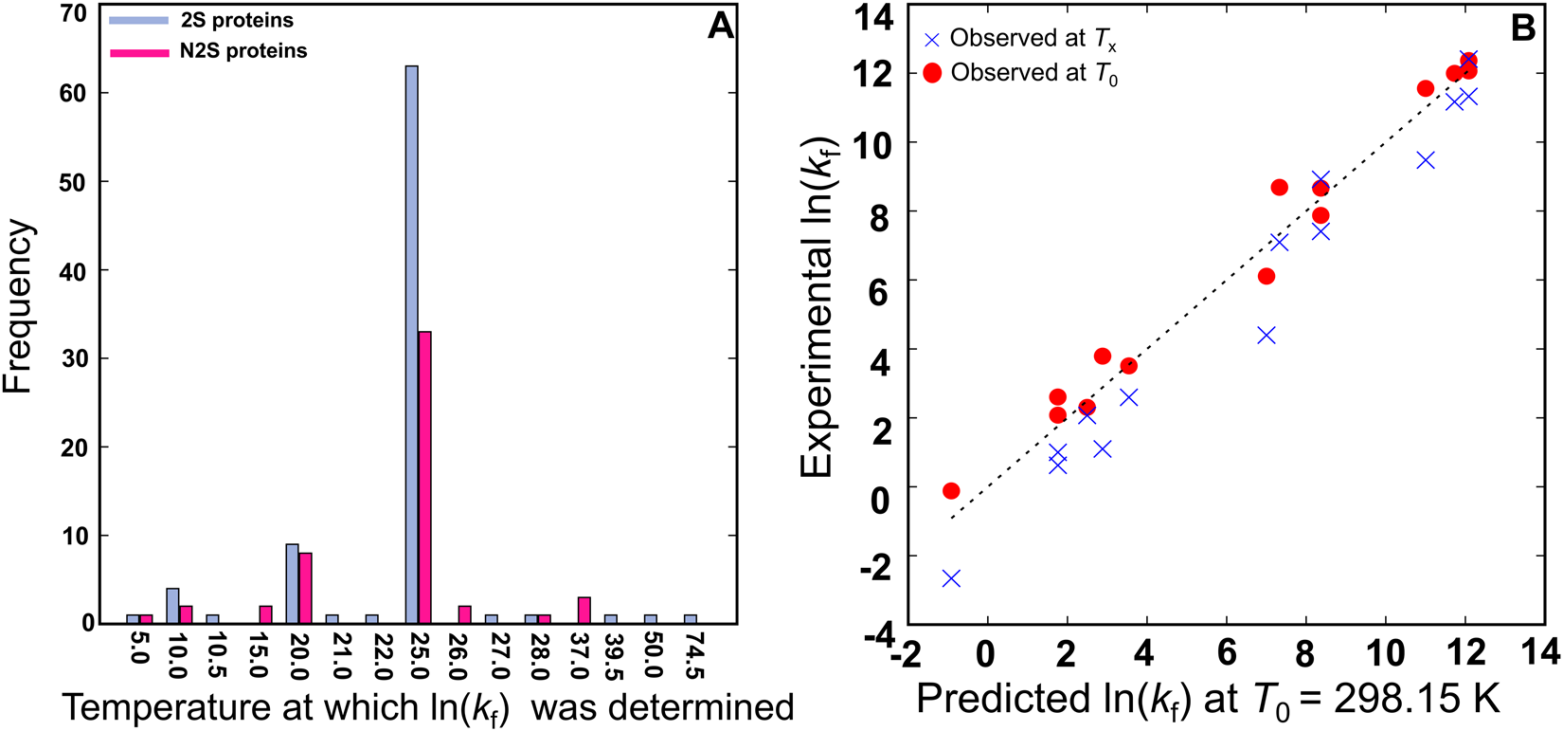
(**A**) The temperature at which ln(*k*_f_) experimentally determined for 2S and N2S is shown. (**B**) Experimentally observed ln[*k*_f_(*T*_0_)] and predicted ones after temperature correction (red circles) are shown. Observed ln[*k*_f_(*T*_x_)] values are also shown for comparison (blue crosses).

Figure 2A shows a distribution of the temperature at which the ln(*k*_f_) was determined experimentally for the proteins in our dataset. Among the 141 proteins in PFDB, 99 were measured at the standard temperature of *T*_0_ (25 °C (=298.15 K)), but the other 42 (24 2S and 18 N2S proteins) were measured at different temperatures (*T*_x_). The *T*_x_ value ranged from 5 °C to 75 °C. To maintain the consistency of folding temperature in PFDB, we developed a method for temperature correction. The predicted shape of the Eyring plot of a particular protein is determined by two parameters of the folding or unfolding reaction, the activation heat capacity (Δ*C*_p_^‡^) and the temperature (*T*_H_) where the activation enthalpy is zero (see Methods for more details). The predicted logarithmic rate constant at *T*_0_ (298.15 K) is thus given by the following equation:

1 $${\rm{l}}{\rm{n}}[k({T}_{0})]\,=\,{\rm{l}}{\rm{n}}[k({T}_{{\rm{x}}})]+[1+\frac{{\rm{\Delta }}{C}_{{\rm{p}}}^{\ddagger }}{R}]{\rm{l}}{\rm{n}}(\frac{{T}_{0}}{{T}_{{\rm{x}}}})+\frac{{\rm{\Delta }}{C}_{{\rm{p}}}^{\ddagger }}{R}[(\frac{1}{{T}_{0}}-\frac{1}{{T}_{{\rm{x}}}})\cdot {T}_{{\rm{H}}}]$$

where *R* is the gas constant, *T*_0_ and *T*_x_ are given by the absolute temperature, and ln[*k*(*T*_x_)] is the logarithmic rate constant measured at *T*_x_; the detailed derivation of Eq. (1) is given in Methods. We assumed that Δ*C*_p_^‡^ is proportional to the heat capacity change (Δ*C*_p_) of the equilibrium protein unfolding. The Δ*C*_p_ is approximately proportional to the protein chain length in the PDB structure (*L*_PDB_) and empirically given by^24^:

2 $${\rm{\Delta }}{C}_{{\rm{p}}}=0.062\cdot {L}_{\text{PDB}}-0.53\,[{\rm{k}}{\rm{J}}{\rm{/}}\text{mol}{\rm{/}}{\rm{K}}]$$

Now, it follows that:

3 $${\rm{\Delta }}{C}_{{\rm{P}}}^{\ddagger }=\beta \cdot {\rm{\Delta }}C{\rm{p}}=\beta ({\rm{0.062}}\cdot {L}_{\mathrm{PDB}}-{\rm{0.53}})\,[\mathrm{kJ}/\mathrm{mol}/K]$$

where *β* is a proportionality constant. Therefore, once we have reasonable estimates of *T*_H_ and *β*, we can evaluate ln[*k*(*T*_0_)] from ln[*k*(*T*_x_)] and *T*_x_ by Eqs (1) and (3). It is worth mentioning that Eq. 2 is an empirical one, and theoretically, the Δ*C*_p_ diminishes to zero when *L*_PDB_ tends to zero. A regression equation between Δ*C*_p_ and *L*_PDB_ with the zero intercept has thus also been reported in the original literature as given by Δ*Cp* = 0.058 *∙* *L*_PDB_^24^. Whether we used this equation or Eq. 2, the results of temperature correction were essentially identical for the proteins in our dataset, where *L*_PDB_ ≥ 34.

#### Temperature correction for folding

We introduced the temperature corrections into the proteins whose *k*_f_ values were measured at a temperature other than the standard temperature (298.15 K). First, we found that the Eyring plot or the equivalent plot of folding was well described in 14 2S proteins and 3 N2S proteins; the *k*_f_ values were measured at every few degrees absolute from ~280 K to ~320 K for most of these proteins^25–41^. Both the *T*_H_ and *β* values for folding kinetics, *T*_Hf_ and *β*_f_, respectively, were more or less common among the different 2S proteins (Table 2) and also among the different N2S proteins (Table 3), except for two 2S proteins (1K9Q^40^ and 1PIN^41^), for which −Δ*C*_p_^‡^ for folding was larger than Δ*C*_p_. Therefore, we employed the 12 2S proteins except for these two and the 3 N2S proteins, and from their Eyring plots, we calculated the *T*_Hf_ and Δ*C*_pf_^‡^. Examples of the Eyring plot for three proteins (1APS^34^, 1D6O^35^, and 1AVZ^37^) are shown in Figure S1. For folding kinetics, the Eyring plot is convexed, and hence, *T*_Hf_ corresponds to the temperature of the maximum point in the Eyring plot. The Δ*C*_pf_^‡^ is given by the curvature of the Eyring plot, and the *β*_f_ was thus evaluated by *β*_f_ = Δ*C*_pf_^‡^/Δ*C*_p_, where Δ*C*_p_ was obtained by Eq. (2); Δ*C*_pf_^‡^ and *β*_f_ are negative because the Eyring plot is convexed. The *T*_Hf_ and *β*_f_ values thus obtained were averaged for the 12 2S proteins and for the 3 N2S proteins (Tables 2 and 3). The *T*_Hf_ and *β*_f_ values thus obtained are 315 ± 1 (standard error estimate) K and −0.62 ± 0.03 for the 2S proteins, and 305 ± 4 K and −0.75 ± 0.07 for the N2S proteins.

**Table 2:** List of proteins used to estimate *T*_Hf_ and *β*_f_ for two-state proteins.

PDB	*L*_PDB_	Temp. (K)	Δ*H*^‡^ (kJ/mol)	${\rm{\Delta }}{{\boldsymbol{C}}}_{{\bf{p}}{\bf{f}}}^{\ddagger }$ (kJ/mol/K)	*T*_Hf_ (K)	Δ*C*_p_ (kJ/mol/K)	*β*_f_
1APS^34^	98	301.15	40.70	−2.57	316.99	5.55	−0.46
1D6O^35^	107	298.15	48.53	−2.80	315.46	6.10	−0.46
1E0G^28^	48	298.15	28.45	−1.76	314.34	2.45	−0.72
1HDN^30^	85	293.15	86.10	−3.22	319.89	4.74	−0.68
2VH7^29^	94	301.15	23.60	−2.48	310.67	5.30	−0.47
3CI2^39^	64	298.00	53.55	−2.05	324.12	3.44	−0.60
1EHB^62^	82	298.15	42.40	−3.60	309.93	4.55	−0.79
1CSP^38^	67	298.15	31.60	−2.70	309.85	3.62	−0.74
1AVZ^37^	57	293.00	43.09	−1.86	316.20	3.00	−0.62
1SHG^36^	57	298.00	37.00	−2.30	314.09	3.00	−0.77
1HCD^31^	118	293.15	57.74	−4.39	306.29	6.79	−0.65
2JMC^25^	77	298.15	45.00	−2.20	318.60	4.24	−0.52
Mean ± SE					314.70 ± 1.44		−0.62 ± 0.03

**Table 3:** List of proteins used to estimate *T*_Hf_ and *β*_f_ for non-two-state proteins.

PDB	*L*_PDB_	Temp. (K)	Δ*H*^‡^ (kJ/mol)	${\boldsymbol{\Delta }}{{\boldsymbol{C}}}_{{\bf{p}}{\bf{f}}}^{{\boldsymbol{\ddagger }}}$ (kJ/mol/K)	*T*_Hf_ (K)	Δ*C*_p_ (kJ/mol/K)	*β*_f_
2CRO^26^	65	293.15	40.70	−3.05	310.50	3.50	−0.87
1PGB^32^	56	298.15	16.80	−1.90	306.99	2.94	−0.64
1L63^33^	162	285.15	92.05	−6.84	298.61	9.51	−0.72
Mean ± SE					305.369 ± 3.526		−0.746 ± 0.067

For the proteins whose *T*_Hf_ and Δ*C*_pf_^‡^ were not available directly, we employed Eqs (1) and (3) to predict ln[*k*_f_(*T*_0_)] by assigning the *T*_Hf_ and *β*_f_ values to *T*_H_ and *β* in the equations. However, for the proteins whose *T*_Hf_ and Δ*C*_pf_^‡^ were available (1E0G^28^, 1HDN^30^, 2VH7^29^, 1EHB^27^, 1HCD^31^, and 2CRO^26^), we directly calculated the ln[*k*_f_(*T*_0_)] values by Eq. (1). To distinguish ln[*k*_f_(*T*_0_)] predicted by using the averaged *T*_Hf_ and *β*_f_ and that directly calculated by Eq. (1) with the known *T*_Hf_ and Δ*C*_pf_^‡^, the latter values are indicated in boldface type in our dataset. It should be also noted that the above *T*_Hf_ and *β*_f_ estimates were based on the folding data of the proteins from mesophilic organisms, and hence some care may be required when applied to the thermophilic proteins.

Next, we compared predicted ln[*k*_f_(*T*_0_)] after the temperature correction with the experimentally observed ln[*k*_f_(*T*_0_)]. For 9 2S and 5 N2S proteins (Table 4), which were not included in those used for estimating *T*_Hf_ and *β*_f_, the experimental ln(*k*_f_) was available at both *T*_0_ and *T*_x_. We thus applied the temperature correction to the ln[*k*_f_(*T*_x_)] values using the above *T*_Hf_ and *β*_f_, and compared predicted ln[*k*_f_(*T*_0_)] with the experimentally observed ln[*k*_f_(*T*_0_)]. From Fig. 2B, the predicted ln[*k*_f_(*T*_0_)] values show good agreement with the experimentally observed ones, showing the validity of our temperature correction. Although the number of data points used for this analysis is not very large (only 14 proteins), it may be enough to suggest that the temperature corrections have improved the quality of the database of protein folding.

**Table 4:** List of Proteins used for predicting ln(*k*_f_) at 25 °C.

PDB	ln[*k*_f_(*T*_x_)]	*T*_x_ (K)	ln[*k*_f_(*T*_0_)] observed	ln[*k*_f_(*T*_0_)] predicted
1FNF^63^	−2.66	278.15	−0.92	−0.12
1IMQ^13,43^	7.09	283.15	7.33	8.69
1K9Q^40,44^	8.92	311.15	8.37	8.67
1K9Q^40,44^	7.41	351.15	8.37	7.87
1RFA^45^	4.40	281.15	7.00	6.11
1SS1^46^	12.41	323.15	12.08	12.07
1SS1^46^	11.33	283.15	12.08	12.37
1U4Q^47,48^	9.48	283.15	11.00	11.56
2WXC^49,50^	11.17	283	11.73	12.00
**1BNI** ^51^	**2**.**07**	**318**.**15**	**2**.**50**	**2**.**31**
1DWR* ^64, 65^	**1**.**10**	**281**.**15**	**2**.**88**	**3**.**79**
**1NFI** ^66^	**1**.**00**	**288**.**15**	**1**.**76**	**2**.**08**
**1NFI** ^66^	**0**.**62**	**283**.**15**	**1**.**76**	**2**.**60**
**1EKG** ^52^	**2**.**60**	**288**.**15**	**3**.**54**	**3**.**51**

*T_0_ for 1DWR was 299.15 K (26 °C).

Normal font and bold, respectively, represent the 2S and N2S proteins.

Denaturant *m* values, the dependence of the free energy of unfolding on denaturant concentration, are well correlated with the Δ*C*_p_ of unfolding^42^. Therefore, we can reasonably assume that *β*_f_ is equivalent to −*β*_T_ for 2S proteins. Therefore, for the 2S proteins for which the *β*_T_ is available, we also calculated the ln[*k*_f_(*T*_0_)] values by assigning the *T*_Hf_ and −*β*_T_ values to *T*_H_ and *β* in Eqs (1) and (3). The ln[*k*_f_(*T*_0_)] values thus obtained are also listed in PFDB and indicated in italic type to distinguish them from those (in roman type) predicted on the basis of *T*_Hf_ and *β*_f_. As seen from the PFDB dataset, these two types of predicted ln[*k*_f_(*T*_0_)] are reasonably coincident with each other.

#### Temperature correction for unfolding

We introduced the temperature corrections into the proteins whose *k*_u_ values were measured at a temperature other than the standard temperature (298.15 K), and the *T*_H_ and *β* values for unfolding kinetics, *T*_Hu_ and *β*_u_, respectively, were required for temperature correction. For unfolding kinetics, the Eyring plot is usually concaved with a positive *β*_u_. For 2S proteins, there is only a single transition state between U and N with a *β*_f_ of −0.62 ± 0.03, and we can reasonably assume that *β*_u_ = 1 + *β*_f_. Therefore, we find that *β*_u_ = 0.38 ± 0.03. For N2S proteins, this simple relationship may not hold, because of a contribution from an intermediate (I) state. For the N2S proteins, however, (1 − *β*_T_) is expected to be equivalent to *β*_u_, because *β*_T_ represents the relative position of the transition state between U and N in terms of the denaturant *m* values. The *β*_T_ was reported for 38 N2S proteins in PFDB, and their average was estimated at 0.79 ± 0.02, and hence *β*_u_ = 0.21 ± 0.02 for N2S proteins; 1FTG was excluded in this calculation because the I state was mostly off-pathway in this protein.

**Figure 3:**
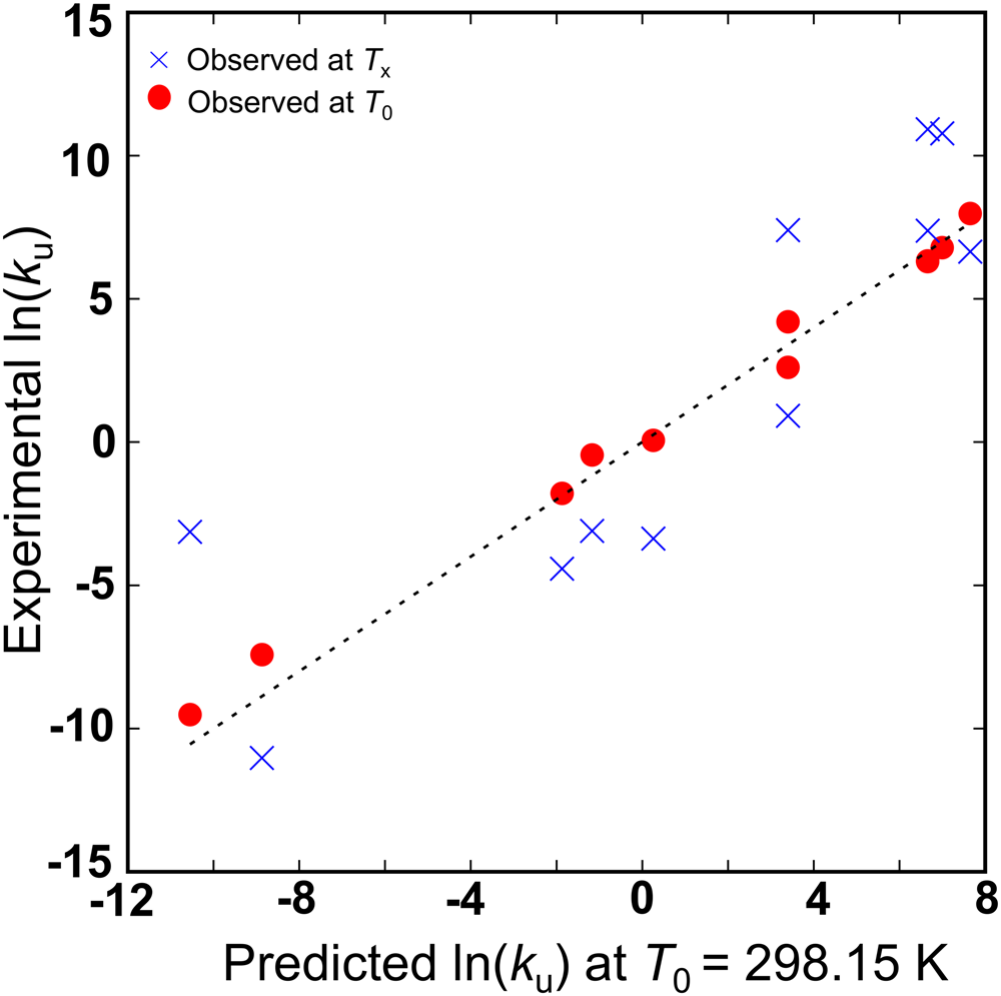
Experimentally observed ln[*k*_u_(*T*_0_)] and predicted ones after temperature correction (red circles) are shown. Observed ln[*k*_u_(*T*_x_)] values are also shown for comparison (blue crosses).

The *T*_Hu_ corresponds to the temperature of the minimum point of the Eyring plot, but this is usually located at far below an observable temperature range of unfolding kinetics, leading to a large error in estimation of *T*_Hu_ due to a long extrapolation along temperature. Furthermore, the Eyring plot of unfolding is not available for many of the proteins used above for estimation of *T*_Hf_ and *β*_f_. Therefore, we had to use a different way to estimate *T*_Hu_. We thus chose 6 2S proteins (1IMQ^13,43^,1K9Q^40,44^, 1RFA^45^, 1SS1^46^, 1U4Q^47,48^, and 2WXC^49,50^) and 3 N2S proteins (1BNI^51^, 1EKG^52^, and 1ENH^53^), for which the experimental ln(*k*_u_) is available at both *T*_0_ and *T*_x_ (Table 5). First, we assumed appropriate *T*_Hu_ values (e.g., 200 K and 150 K) for 2S and N2S proteins, and assigned these *T*_Hu_ values and the above *β*_u_ values to *T*_H_ and *β* in Eqs (1) and (3) to calculated tentative predictions of ln[*k*_u_(*T*_0_)] for 2S and N2S proteins. Then, the *T*_Hu_ values were gradually increased or decreased until the root-mean-square deviation between the experimentally observed ln[*k*_u_(*T*_0_)] and the predicted ln[*k*_u_(*T*_0_)] values was minimized. The optimized *T*_Hu_ values thus obtained were 224 K and 119 K for the 2S and N2S proteins, respectively. Figure 3 shows a comparison between the experimental ln[*k*_u_(*T*_0_)] values and those predicted by using the above *T*_Hu_ and *β*_u_ values, which indicates a reasonable coincidence between the experimental and predicted values.

**Table 5:** List of proteins used for predicting ln(*k*_u_) at 25 °C.

PDB	ln[*k*_u_(*T*_x_)]	*T*_x_ (K)	ln[*k*_u_(*T*_0_)] observed	ln[*k*_u_(*T*_0_)] predicted
1IMQ^13,43^	−4.42	283.15	−1.87	−1.79
1K9Q^40,44^	10.92	351.15	6.66	6.30
1K9Q^40,44^	7.38	311.15	6.66	6.33
1RFA^45^	−3.10	281.15	−1.17	−0.45
1SS1^46^	7.40	323.15	3.40	4.20
1SS1^46^	0.92	283.15	3.40	2.61
1U4Q^47,48^	−3.37	298.15	0.26	0.06
2WXC^49,50^	6.65	283	7.65	7.98
**1BNI** ^51^	**−3**.**13**	**318**.**15**	**−10**.**55**	**−9**.**51**
**1EKG** ^52^	**−11**.**02**	**288**.**15**	**−8**.**87**	**−7**.**42**
**1ENH** ^53^	**10**.**78**	**325**.**3**	**7**.**00**	**6**.**79**

Normal font and bold, respectively, represent the 2S and N2S proteins.

For the proteins whose *T*_Hu_ and Δ*C*_pu_^‡^ were not available directly, we thus employed Eqs (1) and (3) to predict the ln[*k*_u_(*T*_0_)] by assigning the *T*_Hu_ and *β*_u_ values to *T*_H_ and *β* in the equations. However, for the proteins whose *T*_Hu_ and Δ*C*_pu_^‡^ were available (1EHB^27^ and 1HCD^31^), we directly calculated the ln[*k*_u_(*T*_0_)] values by Eq. (1). To distinguish the ln[*k*_u_(*T*_0_)] predicted by using the optimized *T*_Hu_ and *β*_u_ and that directly calculated by Eq. (1) with the known *T*_Hu_ and Δ*C*_pu_^‡^, the latter values are indicated in boldface type in our dataset.

For the 2S proteins for which the *β*_T_ is available, we also calculated the ln[*k*_u_(*T*_0_)] values by assigning the *T*_Hu_ and (1 − *β*_T_) values to *T*_H_ and *β* in Eqs (1) and (3). The ln[*k*_u_(*T*_0_)] values thus obtained are also listed in PFDB and indicated in italic type to distinguish them from those (in roman type) predicted on the basis of *T*_Hu_ and *β*_u_. As seen from the PFDB dataset, these two types of predicted ln[*k*_u_(*T*_0_)] are reasonably coincident with each other.

### Availability of PFDB

As a user-friendly database, PFDB is freely available at http://lee.kias.re.kr/~bala/PFDB. The database main page contains the following options: HOME, N2S, 2S, DOWNLOAD DATASET, and CONTACT. Our dataset can be downloaded by clicking the “DOWNLOAD DATASET” button.

## Conclusions

In this study, we have constructed PFDB, a systematically compiled standardized database of protein folding kinetics. It is currently the most updated one with the highest number of unique entries. The quality of the dataset has been improved significantly by our temperature correction method. Therefore, our dataset can be used as a standard for developing and testing future predictive and theoretical studies of protein folding kinetics.

## Methods

### Construction of the AG dataset

The most recent datasets of protein folding kinetics are ACPro^19^ and the Garbuzynskiy dataset^17^. Prior to the filtering processes shown below, the ACPro dataset contained 126 proteins. Among these, we weeded out proteins with less than 34 residues (1PGB (41–56), 1L2Y and 3M48), proteins with disulfide bonds (2HQI, 1HEL, 1E65 and 1HMK), proteins with a covalently-bound prosthetic group (1YCC, 1YEA, 256B and 1HRC), proteins with irrelevant rate constants (*i*.*e*., the rate constant for formation of an intermediate instead of the actual folding rate constant (*k*_f_) for a few proteins (1AON, 1BD8 and 1JON)), and proteins whose *k*_f_ was reported in the presence of denaturant (1QOP chain B). In the case of ileal lipid binding protein, the actual folding experiment was performed on the rat protein, but its PDB coordinates were not available at the time of our database creation. Instead, the reported PDB ID of 1EAL is the pig protein that is of 71.1% sequence identity with the rat protein. Since the exact PDB coordinates were not available, we excluded this protein as well as another protein without experimental references (1PSF). Furthermore, 6 proteins had duplicate entries (1NTI–2FDQ, 1SRL–1FMK, 1BF4–1BNZ, 1POH–2HPR, 1O6X–1PBA and 1EAL–2EAL) which we corrected. These filtering processes resulted in the reduction of the size of the ACPro dataset from 126 to 102 proteins. We then applied the same filtering scheme to the Garbuzynskiy dataset (107 proteins) where we weeded out proteins with less than 34 residues (1L2Y, 1T8J, 1PGB (41–56), and the 3rd entry in the Garbuzynskiy dataset), proteins with irrelevant rate constants (1AON and 1BD8), the protein 1EAL (the reason is given above), and a protein with a covalently-bound prosthetic group (256B). This change reduced the size of the Garbuzynskiy dataset from 107 to 99 proteins. When we compared the updated Garbuzynskiy (99 proteins) and ACPro (102 proteins) datasets, 6 unique proteins (1IFC, 1CBI, 1IGS, 1OPA, 2MYO and 3H08) were identified in the Garbuzynskiy dataset. Therefore, we added these 6 proteins to the ACPro dataset, and collectively named it the AG dataset (108 proteins).

### Data collection and construction of PFDB

We manually collected the data of protein folding and unfolding kinetics by extensive literature search. Then we compared our collected data with those of the AG dataset. We carefully examined the data of each entry of the AG dataset, and when newer updated data did not exist, the data of that entry were included as such in our dataset of PFDB, otherwise replaced by the updated data. Finally, we added the data of 33 new proteins into the PFDB from our own collection. Of these 33 proteins, 19 are 2S proteins (1DKT, 1FGA, 1IO2, 1KDX, 1NFI,1QAU, 1RG8, 2BKF, 2GA5, 2J5A, 2JMC, 2LLH, 2L6R, 2WQG, 3O48, 3O49, 3O4D, 3ZRT (N-terminal), and 3ZRT (C-terminal)) with the remaining 14 being N2S proteins (1DWR, 1EKG, 1FA3, 1HRH, 1OKS, 1THF, 1UCH, 2BJD, 2FS6, 2KDI, 2KLL, 2X7Z, 3BLM, and 5L8I).

For 4 proteins (1RA9, 1B9C, 1FA3, and 2PQE), the presence of multiple parallel pathways of folding has been reported^54–56^, and the *k*_f_ value was obtained by averaging the rate constant values along the individual pathways:

4 $${{k}}_{{\rm{f}}}=\sum \,_{{i}=1}^{{n}}{{f}}_{{i}}{{k}}_{{i}}$$

where *f*_*i*_ and *k*_*i*_ are the fractional amplitude and the observed rate constant, respectively, for the *i*^th^ pathway of folding, and the ln(*k*_f_) values thus obtained are listed in our dataset.

The ln(*k*_f_), ln(*k*_I_) and ln(*k*_u_) values listed in PFDB are those in the absence of denaturant, usually obtained by linear extrapolation of the logarithmic rate constants along molar denaturant concentration. However, for 5 N2S proteins (1PHP (1–175)^57^, 1PHP (186–394)^58^, 1L63^59^, 1HNG^60^, and 1TTG^61^), the equilibria and kinetics of folding and unfolding were analyzed in terms of denaturant activity rather than the molar concentration. Whether we use the activity or the concentration in our calculation seriously affects the ln(*k*_u_) estimation, because a long extrapolation from high concentrations of denaturant back to the native condition is required. To keep consistency of our dataset, we used the linear extrapolation along the molar concentration, as far as such data were available, to estimate the ln(*k*_u_).

### Derivation of Eq (1) for the temperature correction

In this study, we introduced a method for temperature correction, which gives the folding and unfolding rate constants at 25 °C (*k*(*T*_0_) where *T*_0_ = 298.15 K) for a protein whose rate constant at any temperature (*T*_x_) is known. The following section will describe the derivation of Eq. (1).

According to the Eyring–Kramers equation^20^, we find that:

5 $${\rm{l}}{\rm{n}}(\frac{k}{T})=C-\frac{1}{RT}[{\rm{\Delta }}{H}^{\ddagger }({T}_{{\rm{H}}})-T{\rm{\Delta }}{S}^{\ddagger }({T}_{{\rm{H}}})+{\rm{\Delta }}{C}_{{\rm{p}}}^{\ddagger }\cdot \{T-{T}_{{\rm{H}}}-T\cdot \,{\rm{l}}{\rm{n}}(\frac{T}{{T}_{{\rm{H}}}})\}]$$

where Δ*H*^‡^(*T*_H_) and Δ*S*^‡^(*T*_H_) are the activation enthalpy and the activation entropy, respectively, at a reference temperature *T*_H_, and ${\rm{\Delta }}{C}_{{\rm{p}}}^{\ddagger }$ is the activation heat capacity; we assume that ${\rm{\Delta }}{C}_{{\rm{p}}}^{\ddagger }$ is a constant independent of temperature (*T*). When we set *T*_H_ to the temperature where Δ*H*^‡^ is zero, i.e., the maximum or minimum point of the Eyring plot, Eq. (5) is rewritten as:

6 $${\rm{l}}{\rm{n}}(\frac{k}{T})={C}_{2}-\frac{{\boldsymbol{\Delta }}{C}_{{\rm{p}}}^{\ddagger }}{RT}\cdot [T-{T}_{{\rm{H}}}-T\cdot \,{\rm{l}}{\rm{n}}(\frac{T}{{T}_{{\rm{H}}}})]$$

where *C*_2_ is a temperature-independent constant (*C*_2_ = *C* + Δ*S*^‡^(*T*_H_)/*R*). When ${\rm{\Delta }}{C}_{{\rm{p}}}^{\ddagger }$ and the Δ*H*^‡^(*T*_a_) at a particular temperature (*T*_a_) are known, *T*_H_ is simply given by *T*_H_ = [*T*_a_ − Δ*H*^‡^(*T*_a_)/${\rm{\Delta }}{C}_{{\rm{p}}}^{\ddagger }$]. From Eq. (6), we can obtain the temperature dependence of ln(*k*/*T*), once we have *T*_H_ and Δ*C*_p_^‡^. The difference in ln(*k*/*T*) between *T*_0_ (=298.15 K) and *T*_x_ is thus given by:

7 $$\mathrm{ln}[\frac{k({T}_{0})}{{T}_{0}}]-\,\mathrm{ln}[\frac{k({T}_{{\rm{x}}})}{{T}_{{\rm{x}}}}]=\frac{{\rm{\Delta }}{C}_{{\rm{p}}}^{\ddagger }}{R}\cdot [\frac{{T}_{{\rm{H}}}}{{T}_{0}}-\frac{{T}_{{\rm{H}}}}{{T}_{{\rm{x}}}}+\,\mathrm{ln}(\frac{{T}_{0}}{{T}_{{\rm{x}}}})]$$

Therefore, we obtain Eq. (1).

## Supplementary information

Supplementary information
