# Supplementary material for: PFDB: A standardized protein folding database with temperature correction
Source: Sci Rep. 2019 Feb 7;9:1588. doi: 10.1038/s41598-018-36992-y (PMC6367381; doi:10.1038/s41598-018-36992-y)

**PFDB: A standardized protein folding database with temperature correction**

Balachandran Manavalan^1^, Kunihiro Kuwajima^1,2,3,*^ and Jooyoung Lee^1,*^

^1^ School of Computational Sciences, Korea Institute for Advanced Study (KIAS), Seoul, Korea, ^2^CPIS, the Graduate University for Advanced Studies (Sokendai), Hayama, Japan, and ^3^Department of Physics, School of Science, the University of Tokyo, Tokyo, Japan.

*To whom correspondence should be addressed.

Kunihiro Kuwajima: kuwajima@ims.ac.jp; Jooyoung Lee: jlee@kias.re.kr

**Figure S1**. Examples of the Eyring plot for three proteins, 1APS, 1D6O, and 1AVZ, are shown in A, B, and C, respectively. All these data points were extracted from their respective literatures^34,35,37^ and fitted according to the Eq. 6. Our estimated ${\text{∆}\text{C}}_{\mathrm{pf}}^{\ddagger}$ (kJ/mol/K) and *T*_Hf_ (K) values shown in the figures are in reasonable agreement with the values reported in their respective literatures.


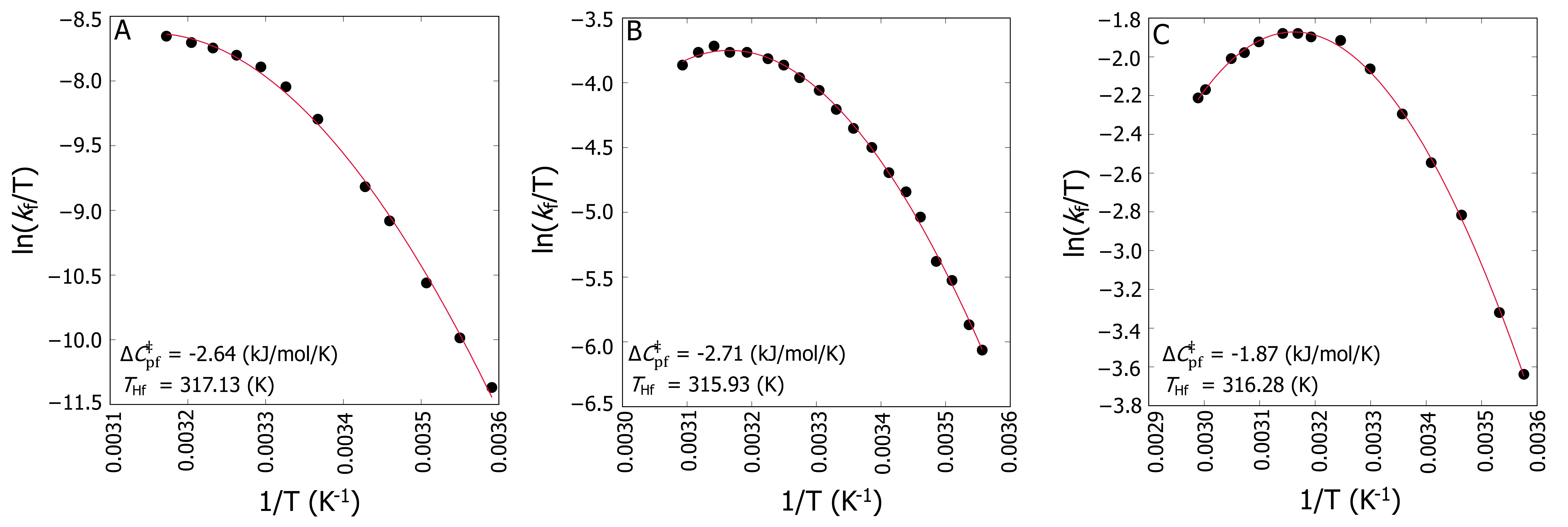

Supplement: Supplementary file 1 — Supplementary information [file 41598_2018_36992_MOESM1_ESM.docx]
